# Primary and secondary cases in *Escherichia coli *O157 outbreaks: a statistical analysis

**DOI:** 10.1186/1471-2334-9-144

**Published:** 2009-08-28

**Authors:** Kate G Snedeker, Darren J Shaw, Mary E Locking, Robin J Prescott

**Affiliations:** 1 Department of Population Medicine and Centre for Public Health and Zoonoses, Ontario Veterinary College, University of Guelph, Guelph, Ontario, N1G 2W1, Canada; 2 Centre for Population Health Studies, The University of Edinburgh, Teviot Place, Edinburgh, EH8 9AG, UK; 3 Veterinary Clinical Sciences, Royal (Dick) School of Veterinary Studies, The University of Edinburgh, Easter Bush Veterinary Centre, Roslin, Midlothian, EH25 9RG, UK; 4 Health Protection Scotland, Clifton Place, Glasgow, G3 7LN, UK

## Abstract

**Background:**

Within outbreaks of *Escherichia coli *O157 (*E. coli *O157), at least 10–15% of cases are thought to have been acquired by secondary transmission. However, there has been little systematic quantification or characterisation of secondary outbreak cases worldwide. The aim of this study was to characterise secondary outbreak cases, estimate the overall proportion of outbreak cases that were the result of secondary transmission and to analyse the relationships between primary and secondary outbreak cases by mode of transmission, country and median age.

**Methods:**

Published data was obtained from 90 confirmed *Escherichia coli *O157 outbreaks in Great Britain, Ireland, Scandinavia, Canada, the United States and Japan, and the outbreaks were described in terms of modes of primary and secondary transmission, country, case numbers and median case age. Outbreaks were tested for statistically significant differences in the number of ill, confirmed, primary and secondary cases (analysis of variance and Kruskal-Wallis) and in the rate of secondary cases between these variables (Generalised Linear Models).

**Results:**

The outbreaks had a median of 13.5 confirmed cases, and mean proportion of 0.195 secondary cases. There were statistically significant differences in the numbers of ill, confirmed, primary and secondary cases between modes of primary transmission (p < 0.021), and in primary and secondary cases between median age categories (p < 0.039) and modes of secondary transmission (p < 0.001).

Secondary case rates differed statistically significantly between modes of secondary and primary transmission and median age categories (all p < 0.001), but not between countries (p = 0.23). Statistically significantly higher rates of secondary transmission were found in outbreaks with a median age <6 years and those with secondary transmission via person to person spread in nurseries. No statistically significant interactions were found between country, mode of transmission and age category.

**Conclusion:**

Our analyses indicated that ~20% of *E. coli *O157 outbreak cases were the result of secondary spread, and that this spread is significantly influenced by age and modes of primary and secondary transmission, but not country. In particular, the results provide further data emphasising the importance of simple but effective preventive strategies, such as handwashing, that can reduce the risk of secondary spread, particularly amongst young children in nurseries.

## Background

*Escherichia coli *O157 (*E. coli *O157) infections are a major contributor to severe infectious gastrointestinal illness in the developed world, resulting in 150–250 cases reported annually in Scotland [1] and 2500–4500 cases in the United States[2]. Infection can lead to child mortality[3] as well as financial costs of millions of pounds in medical expenses and lost productivity[4,5]. Most cases appear to be sporadic, in that they are not epidemiologically linked to other cases, but outbreaks have accounted for between 2% and 28% of infections [6-8]. Outbreaks have ranged from 1 to 501 confirmed cases [9] and 2 to > 6000 ill cases[10], with the severity of outcomes ranging from just 3% of ill persons hospitalised and no deaths[11] to 26% hospitalised[12] and 17 deaths[13].

*E. coli *O157 infection can be acquired directly from an initial or point source(s) of the bacteria, whether it is an infected animal, animal faeces, contaminated food or water [14]. Though food, water and person to person spread are long recognised modes of transmission [15], in more recent years, infection has been more directly linked to animal and environmental exposures[16,17]. Secondary transmission from an infected person, directly or indirectly[18] to another person, also occurs [19,20] with sources suggesting between 10–20% of outbreak cases are acquired by secondary transmission [20-23].

Secondary spread also occurs between household members, with age an important factor in determining those likely to be infected – because they have immature immune systems and are not yet skilled in thorough hygiene practices, young children are most likely to both transmit to and be infected by, household contacts [24]. This association between children and infection is reflected in other locations where outbreaks have the potential to involve secondary cases, including nurseries[25], petting zoos[26] and swimming areas [18,27]. This association is particularly important because of age-related morbidity and mortality[19]. Furthermore, people infected via secondary transmission appear to have a similar likelihood of severe outcomes (e.g. haemolytic uraemic syndrome, HUS) as those infected by direct exposure[6].

Despite differences between countries in epidemiologically relevant characteristics like population density and land use, reported secondary case rates of 10–20% overall[22] and >50% for individual outbreaks[28] in multiple countries indicate that secondary spread is important in many situations. An examination of the relationship between primary and secondary outbreak cases, in particular any differences between modes of primary and secondary transmission and countries, is therefore warranted. While some countries do report population based figures for secondary spread[6,24], there has been little systematic quantification of secondary outbreak cases, particularly at an international level. Prior analyses have generally been purely descriptive [20,25,29], with definitions of primary mode of transmission for surveillance purposes sometimes based upon the mode accounting for most cases, rather than the mode of transmission to the initial case or cases[30,31]. Also, while nursery outbreaks have been mentioned as group with high rates of secondary infection[20], there have been almost no comparisons between outbreak primary or secondary modes of transmission, or between countries by age.

The aim of this study is therefore to describe and characterise *E. coli *O157 primary and secondary cases in outbreaks; to estimate the proportion of these cases resulting from secondary transmission; and to analyse the relationships between primary and secondary outbreak cases by mode of transmission, country and median age.

## Methods

### Literature Search

Medline^®^, the Web of Knowledge^® ^and archives of publications issued by national public health organizations were searched for reports of *E. coli *O157 outbreaks occurring between 1^st ^January 1982 and 31^st ^December 2006 in Scotland, England and Wales, the United States, Canada, Japan, Ireland, Denmark, Finland, Norway and Sweden. The search terms were "O157", "outbreak", "STEC" and "VTEC". National publications searched were Mortality and Morbidity Weekly (USA), Canadian Communicable Disease Report, (Canada), Communicable Disease Review Weekly and Communicable Disease and Public Health (England & Wales), Health Protection Scotland (HPS) (formerly SCIEH) Weekly Reports, Infectious Agents Surveillance Reports English version (Japan), Epi-Insight (Ireland), EpiNews (Denmark) and Eurosurveillance (European Union).

### Time Period

Published outbreaks from 1982 to mid 2006.

### Definitions

For this study, a confirmed case was defined as a person-infection-episode of *E. coli *O157 infection confirmed by culture of faecal isolates, or serodiagnosis. An ill case was epidemiologically linked to other case(s) within an outbreak, irrespective of microbiological confirmation. Outbreaks were defined as events with two or more epidemiologically related confirmed cases, and were included providing the total number of confirmed primary and secondary cases could be determined (see below).

Routes of transmission in outbreaks were categorised as follows:

1. exposure to a suspect or contaminated food, subdivided into:

○ *dairy products *– *e.g*. milk, cheese, yogurt, ice cream, cheese curds and ice cream or

○ *food *– all other foodstuffs.

2. *water *– exposure to a water supply or to recreational water contaminated by a non-human source

3. *animal contact *– a direct contact or exposure to animals

4. *environmental *– exposure to animal faeces or environments, where direct animal contact was very unlikely

5. *person to person *(p-p)- transmission subdivided by specific setting:

○ *institution *– residential facility in which meals are prepared in a common kitchen eg hospitals, prisons and nursing homes.

○ *nursery *– any children's day-care facility where attendees are generally aged ≤ 5 years

○ *home *– private home.

○ *other *– any other public or commercial location including restaurants, camps and non-residential schools (combined due to low numbers)

6. *water secondary *– exposure to recreational water contaminated by another human

7. *unknown *– the outbreak data or report contains:

○ no identifiable *primary *source/exposures to initial risk factors (*unknown mode of primary transmission)*; or:

○ no identifiable exposure to a *secondary *risk factor such as water contaminated by another person, or another ill or infected person.

For the purposes of this study, each outbreak was categorised by mode of both primary and secondary transmission. New mode of transmission definitions were created because the data included outbreaks from different countries, with many varying definitions of mode of transmissions, and reporting forms. In addition, categories used for surveillance purposes generally defined outbreaks by the predominant mode of transmission, i.e. the mode which accounted for the greatest number of cases, irrespective of whether that was the mode of transmission from the primary source, or the subsequent, mode of secondary transmission via water or person to person spread. Also the original outbreak data did not systematically separate out modes of primary, from secondary, transmission.

The primary mode of transmission was defined as the mode of outbreak transmission involving exposure to the initial source of contamination (routes 1, 2, 3, 4 or 7), whilst the mode of secondary transmission was defined as the mode of transmission accounting for the highest number of secondary cases (as defined below). Where no secondary transmission was reported (N/A), outbreaks were not included in mode of secondary transmission analyses. In outbreaks with more than one suspected mode of secondary transmission, the transmission route of the majority of secondary cases was selected.

Primary and secondary case definitions were also not consistent across publications and countries, or were not included; therefore standard case definitions were established to define the nature of the cases within each outbreak:

○ *primary case *– a person reported as having direct exposure to the suspected initial or point source of *E. coli *O157 through transmission routes 1 – 4, 7 as detailed above.

○ *secondary case *– a person with no reported exposure to the suspected initial or point source, who was judged to have acquired infection by secondary transmission via routes 5, 6 or 7.

A four level variable for median age of outbreak cases was established: < 6 years, 6–16, 17–59 and ≥ 60.

### Statistical Analysis

All analyses were performed using R (Version 2.4.0, R Foundation, 2006), with the exception of the Generalised Linear Model (GLM) analyses, which were performed using SAS^® ^Version 9.1. In analyses, categories with unknown values or containing fewer than five outbreaks were combined or omitted. For analyses involving mode of primary transmission, 'animal contact' and 'environmental' categories were combined, and for those involving mode of secondary transmission, 'N/A' and 'unknown' categories were omitted, and 'p-p institution' and 'p-p other' were combined. For analyses involving country, Sweden, Ireland and Finland were omitted, and Wales and England were combined (Table 1).

**Table 1:** Outbreaks in the descriptive study (n = 90), split by country and modes of transmission

Country		Mode of primary transmission	
	**Outbreaks (%)**		**Outbreaks (%)**

United States	27 (30.0)	**Food**	38 (42.2)
England	23 (25.6)	**Dairy Product**	11 (12.2)
Scotland	16 (17.8)	**Animal Contact**	7 (7.8)
Canada	13 (14.4)	**Water**	6 (6.7)
Japan	6 (6.7)	**Environmental**	2 (2.2)
Sweden	2 (2.1)	**Unknown**	26 (28.9)
Ireland	1 (1.1)		
Finland	1 (1.1)		
Wales	1 (1.1)		

**Mode of secondary transmission**			

		**Outbreaks (%)**	

Person to Person – home		41 (45.6)	
Person to Person – nursery		10 (11.1)	
Water		9 (10.0)	
Person to Person – Institution		4 (4.5)	
Person to Person – Other		3 (3.3)	
Unknown		2 (2.2)	
No secondary cases		21 (23.3)	

The outbreaks included in the descriptive study (n = 90), split by country and modes of primary and secondary transmission. Percentages shown are out of the total outbreaks.

Analysis of Variance (ANOVA) analyses were used to check for statistically significant differences in the log-transformed number of ill, confirmed and primary cases between modes of transmission, countries and age categories, with contrasts used to examine the differences between the individual variable levels. Due to the distribution of the data, Kruskal-Wallis Tests were performed to compare numbers of secondary cases between modes of transmission and country. Fisher's Exact Tests were used to check for significant differences in age categories between modes of transmission and countries. Mann-Whitney Tests were used to test for differences between pairs of variable levels.

Relationships between the rate of secondary cases and modes of transmission, country and median age were explored using univariate GLMs with Poisson errors, with mode of transmission, country or median age category inserted as the explanatory variable, number of secondary cases as the response variable and log transformed number of primary cases as the offset variable. To adjust for overdispersion, a scale parameter, estimated by the square root of deviance/degrees of freedom, was used. Differences between different modes of transmission, countries and age categories were examined using least square mean differences.

Multivariate analyses including the above explanatory variables were run using GLM models to look for potential confounders and interactions between explanatory variables. Model simplification was achieved through the omission of non-statistically significant model terms. In all cases, values of p < 0.05 were considered to be statistically significant, with subscripts used to indicate degrees of freedom.

## Results

### Descriptive summary

**Figure 1:**
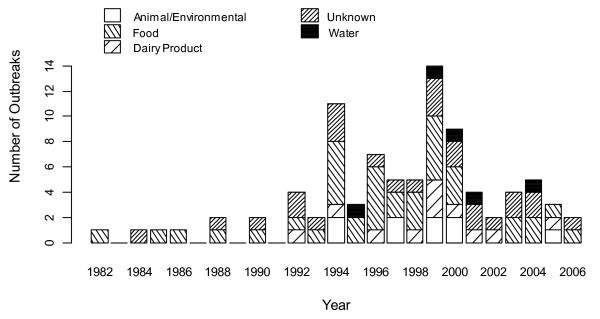
**Outbreaks included in the descriptive study**. Bar plot of outbreaks included in the descriptive study (n = 90) divided by year and mode of primary transmission.

**Figure 2:**
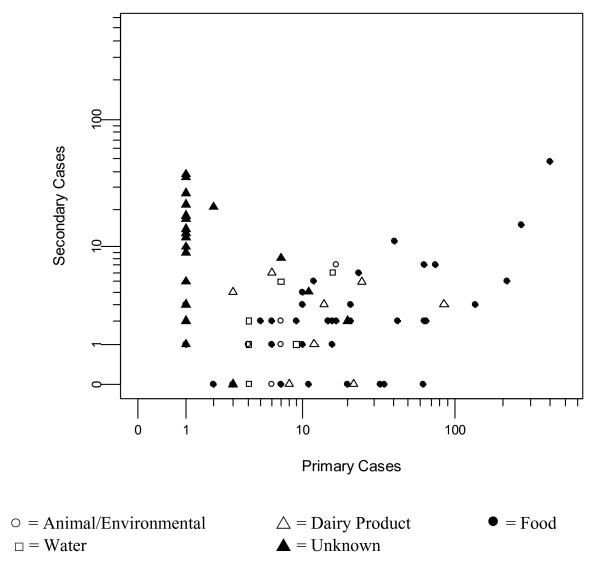
**Number of secondary cases against number of primary cases, by mode of primary transmission**. Number of secondary cases against number of primary cases on a log (1+x) scale, subdivided by mode of primary transmission.

From 1982 to 2006, reports were available for 176 outbreaks. Of these, 90 matched the study inclusion criteria, the majority of which occurred between 1994 and 2000 (Figure 1 and Additional file 1). The outbreaks took place in nine countries, with 30% occurring in the United States (Table 1). The mean proportion of secondary cases for all 90 outbreaks was 0.195 (range 0 to 0.97), and the overall rate of secondary cases per primary case was 0.24. In the 75 outbreaks where median age could be determined, 26% had a median age of < 6 years, 36% 6–16 years, 31% 17–59 years and 7% ≥ 60 years. The highest mean proportion of secondary cases was found in outbreaks with a median age of cases <6 years (0.65: 95% CI = 0.60–0.70) and the lowest in outbreaks with a median age of 17–59 (0.09: 0.07–0.11). The frequency distributions of ill, confirmed, primary and secondary cases were all strongly positively skewed, with median outbreak sizes of 17 ill (range 2 to 738), 13.5 confirmed (range 2 to 501), seven primary (range 1 to 398) and two secondary (range 0 to 48) cases. When the numbers of primary cases in each outbreak were plotted against the number of secondary cases (Figure 2), there was no linear relationship. The majority of outbreaks (81%) have <50 primary cases and <15 secondary cases, with the largest numbers of secondary cases seen in outbreaks with either ≤ 2 or >100 primary cases.

Eighty-six outbreaks were used for the country specific statistical analyses and 75 for median age. Between countries there was a statistically significant difference in the log-transformed number of ill cases (p = 0.033; Table 2), but not in confirmed, primary or secondary cases (p > 0.082). Post-hoc analyses revealed that Japanese outbreaks had higher numbers of ill cases than Canadian, English & Welsh and Scottish outbreaks (p < 0.034), and United States outbreaks had higher numbers of ill cases than English & Welsh outbreaks (p = 0.027, Table 2). A different pattern was seen for the median age analyses with statistically significant differences in the number of (log transformed) primary and secondary cases (p < 0.038), but no differences in number of ill and confirmed cases (p > 0.64, Table 2). Outbreaks with a median age of <6 years had lower numbers of primary cases than outbreaks in all other median age categories (p < 0.025), and higher numbers of secondary cases than outbreaks with a median age of 17–59 (p = 0.007). There was no statistically significant difference between countries in the proportion of outbreaks in each median age category (Fisher's Exact p = 0.74).

**Table 2:** Distribution plots of the number ill, confirmed, primary and secondary cases in the outbreaks

	Ill Cases	Confirmed Cases	Primary Cases	Secondary Cases
**Country**				
United States	25.2 (14.7, 43.1)	14.5 (8.6, 24.6)	6.2 (3.2, 12.2)	2 (2,6)
England & Wales	11.9 (7.9, 17.9)	10.8 (7.5, 15.6)	4.7 (2.8, 7.8)	3 (1,7)
Scotland	14.5 (7.5, 31.7)	14.4 (7.5, 27.6)	9.3 (3.9, 21.8)	2 (0,5)
Canada	13.4 (7.2, 24.9)	9.4 (4.8, 18.7)	5.5 (2.3, 13.4)	2 (1,5)
Japan	52.9 (17.2, 163.3)	43.3 (14.8, 126.4)	23.8 (3.4, 164.2)	3 (0,22)

**Median age**				
< 6 years	13.9 (9.0, 21.5)	10.4 (6.5, 16.7)	2.0 (1.2, 3.4)	6 (3,13)
6–16 years	18.9 (10.2, 35.0)	14.5 (8.5, 25.0)	8.3 (4.2, 16.4)	2 (2,4)
17 – 59 years	20.2 (11.4, 35.7)	13.2 (7.5, 23.1)	10.2 (5.5, 19.0)	2 (0,2)
60+ years	29.5 (9.9, 88.4)	20.7 (11.1, 38.5)	10.9 (1.8, 65.1)	4 (0,10)

**Mode of Primary Transmission**				
Animal/Environmental	6.7 (3.6, 12.7)	6.6 (3.5, 12.5)	5.1 (2.4, 10.7)	1 (0,3)
Dairy products	12.0 (5.6, 25.7)	10.4 (4.9, 22.1)	8.1 (3.5, 18.8)	1 (0,5)
Food	33.8 (21.6, 52.8)	20.9 (13.4, 32.4)	18.1(11.5, 28.4)	2 (1,2)
Water	8.5 (4.5, 16.1)	8.3 (4.2, 16.1)	6.3 (3.5, 11.5)	1.5 (1,6)
Unknown	14.2 (10.5, 19.1)	11.3 (8.2, 15.5)	1.4 (1.0, 2.0)	11 (5,17)

**Mode of Secondary Transmission**				
P-P home	23.8 (15.7, 36.2)	19.8 (13.4, 29.1)	11.9 (7.2, 19.6)	2 (2,5)
P-P nursery	25.8 (18.0, 37.1)	15.7 (10.2, 24.3)	1.4 (0.8, 2.6)	15.5 (9,22)
P-P other	15.5 (5.7, 41.9)	11.2 (5.3, 23.9)	6.0 (1.9, 19.0)	3 (1,10)
Water	11.9 (5.6, 25.5)	9.9 (4.3, 23.0)	1.6 (0.5, 4.9)	5 (3,17)

The geometric mean number of ill, confirmed and primary cases and median number of secondary cases by country (n = 86), median age (75), modes of primary (90) and secondary (67) transmission are shown. For the geometric means, 95% confidence intervals are shown and for the medians, range is given.

Food was the most often cited mode of primary transmission (42%) and environmental the least (2%, Table 1). In 29% of outbreaks the mode of primary transmission was unknown. There were statistically significant differences between modes of primary transmission in log-transformed numbers of ill, confirmed and primary cases and the median number of secondary cases (p < 0.021, Table 2). Post-hoc analyses revealed that outbreaks where food was the primary mode of transmission had higher numbers of ill and primary cases than outbreaks with any other primary mode (p < 0.045), and higher numbers of confirmed cases than outbreaks categorised as animal/environmental or unknown (p < 0.031). Additionally, outbreaks where the primary mode was unknown had lower log-transformed mean numbers of primary cases (p < 0.005) and higher median numbers of secondary cases (p < 0.01) than outbreaks with all other (known) primary modes. There were also statistically significant differences in median age between modes of primary transmission (Fisher's Exact p = 0.004): outbreaks where food was the mode of primary transmission were statistically significantly different from water or unknown outbreaks (p < 0.041).

All 90 outbreaks were considered when describing mode of secondary transmission. The most common mode of secondary transmission was person to person within the home (46%), but 23% of outbreaks did not have identified secondary cases (Table 1). For statistical analyses, only the 67 outbreaks with a known mode of secondary transmission were included. There were statistically significant differences between modes of secondary transmission in the log-transformed numbers of primary and secondary cases (p < 0.003) cases, but not in ill and confirmed cases (p > 0.29, Table 2). Outbreaks where the mode of secondary transmission was person to person spread in a nursery had lower numbers of primary cases (p < 0.046), and higher secondary cases (p < 0.016) than all other outbreaks with person to person modes of secondary transmission. In addition, water outbreaks had lower numbers of primary cases (p < 0.001) and higher secondary cases (p = 0.043) than those where the mode of secondary transmission was person to person in the home. There were also significant differences in median age between modes of secondary transmission (Fisher's Exact p < 0.001).

### Rate of secondary cases in relation to primary cases

**Figure 3:**
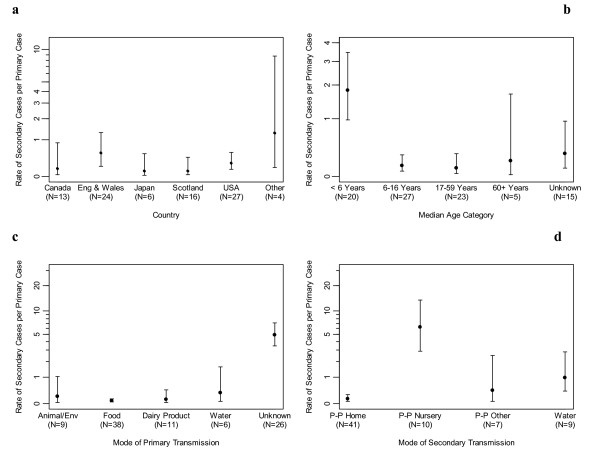
**a – d. Mean rates of secondary outbreak cases per primary case**. Mean (and 95% confidence intervals) rates of secondary outbreak cases per primary case, by a) country, b) median age, c) mode of primary transmission and d) mode of secondary transmission shown on log (1+x) scales. For 3a 'other' encompasses outbreaks from Scandinavia and Ireland, and for 3b the 'unknown' category encompasses outbreaks where the median age is not known.

There was no statistically significant overall difference between countries in the rate of secondary cases to primary cases (p = 0.23, Figure 3a), but there was between median age groups (p < 0.001). Outbreaks with a median age <6 years had a higher rate of secondary cases to primary cases than outbreaks with a median age of 6–16 or 17–59 years (p < 0.001, Figure 3b).

There was also a significant difference between modes of primary transmission in the rate of secondary cases (p < 0.001, Figure 3c). Outbreaks with an unknown mode of primary transmission had a higher rate of secondary cases than outbreaks with all other modes (p < 0.002). When the unknown outbreaks were omitted, there was still a statistically significant difference in secondary case rates (p = 0.030), with water outbreaks having a higher rate than food (p = 0.003).

No statistical analysis of the three-way interaction between mode of primary transmission, country and median age was possible because there were no outbreaks within some mode-country-median age combination categories (e.g. Animal/Environmental-any country-median age ≥ 60, Food-Scotland-median age ≥ 60, Milk-United States – median age other than <6). When all three variables were included in a model without interaction terms, there were statistically significant differences in the rate of secondary cases between modes of primary transmission and median age categories (p < 0.001). Outbreaks in which median age was <6 had higher rates than those of any other age (p < 0.040), and outbreaks with median age 6–16 had higher rates than outbreaks with median age 17–59 (p < 0.026). There were no statistically significant interactions when any two of the above variables were included (p > 0.120). In all instances, the differences in secondary case rates were no longer statistically significant when the outbreaks for which the mode of primary transmission was unknown (p > 0.13) were excluded.

There was a significant difference between modes of secondary transmission in the rate of secondary cases (p < 0.001, Figure 3d). Outbreaks where the mode of secondary transmission was person to person spread in a nursery had higher rates than those where secondary transmission was by person to person spread in the home or other setting, or by water (p < 0.007). Outbreaks where the mode of secondary transmission was water, had higher rates than outbreaks where secondary transmission was person to person spread in the home (p = 0.002).

No analysis could be conducted when all three variables were included with interaction terms. When the interaction terms were removed, there were statistically significant differences in the rate of secondary cases between modes of secondary transmission (p < 0.001) and median age (p < 0.001). Post-hoc analyses revealed that outbreaks where mode of secondary transmission was person to person spread in nurseries or water secondary, had higher rates than those outbreaks where secondary transmission was person to person spread in the home (p < 0.002), Outbreaks with median age <6 had higher rates compared to those with median age 6–16 and 17–59 (p < 0.001).

## Discussion

These results provide what we believe to be the first large-scale, international systematic descriptive and statistical analysis of primary and particularly secondary cases in *E*. coli O157 outbreaks. These analyses indicate that there is a statistically significant relationship between the rate of secondary cases, and modes of primary and secondary transmission, and median age. This reflects the findings of other studies. In contrast, there appears, for example, to be no such relationship between the rate of secondary cases and country.

However, some caution must be taken in interpreting and generalising from these results. Firstly data availability was limited; for example, of the 350 outbreaks in the United States between 1982 and 2006[32], <30 are included in our analyses. In addition, outbreaks in some non-English speaking countries may be under-represented in this study because the majority are not detailed in published English-language reports. Countries also differ in the number of published outbreak reports, with more coming from the United States and England & Wales in particular. The proportions of outbreaks in our study from each country and modes of primary and secondary transmission may therefore not reflect actual proportions. However, the distribution of outbreaks by mode of primary transmission in this study is similar to the proportions in reported outbreaks in the United States, Scotland and England & Wales; food was the mode of transmission in approximately 42% of outbreaks reported in Scotland from 1990–2004 (based on modes provided in annual reports e.g. ref 32), 47% in the United States from 1982–2002, 50% in England & Wales from 1995–2004 and 45% in this study ([32-35]). The 90 outbreaks included in our study, with a geometric mean of 13.5 confirmed cases, were larger than outbreaks in the data sets of all reported outbreaks between 1982 and 2004 in the included countries, such as Scotland (4.1) and United States (5.5). This bias was anticipated since larger outbreaks are more likely to be reported in the literature and thus the results of our study are likely to be more representative of larger outbreaks. We do not, however, know how the inclusion of the smaller outbreaks would influence the rate of secondary cases, as they could be more or less likely to have secondary cases.

In addition, the decision whether to classify a case as primary or secondary was made based upon the information provided in each publication, which means our definition of a secondary case differs from that reported in some outbreak investigations [13,36]. Firstly, it is acknowledged that the categories for primary and secondary mode of transmission were relatively broad categories, and thus some subtlety of information was lost. However, use of the broad categories was standardised, and allowed data to be included from reports which varied in the detail of outbreak description and outbreak or case definitions, and thus maximised the number of studies in the outbreak. In many of these investigations, secondary cases were generally defined as being close and/or family contacts of primary cases[9,36]. In this study, all nursery and water cases other than the case(s) who introduced the infection into the nursery or the water were defined as secondary cases. Thus, the proportion of secondary cases in these outbreaks may be much higher than the proportion calculated when other definitions are used. Also, investigations that included broad-based screening of potential case patients and their contacts, such as those in Japan[37,38], may be more likely to pick up asymptomatic cases. Since it has been suggested that as many as 40% of secondary cases may be asymptomatic[24], the number of secondary cases reported may depend greatly on the level of testing and epidemiological study – although asymptomatic infection is not necessarily acquired by secondary spread [6]. Finally, the inclusion in our analyses of outbreaks where the primary mode of transmission was unknown may have added a degree of uncertainty. However, by defining these outbreaks as a separate category, they could be included in analyses of secondary transmission; the increase in the number of included outbreaks improving the statistical power of the analyses. Additionally, since outbreaks with an unknown mode of transmission trended to involve significantly higher numbers of secondary cases and lower numbers of primary cases, as well as a significantly lower median age than outbreaks with other primary modes, excluding these outbreaks would have further biased the statistical analyses. It was also then possible to run analyses excluding these outbreaks to assess their affect on the statistical analyses. Indeed they did appear to have a significant affect on analyses, as – not surprisingly – outbreaks where the primary mode of transmission was unknown tended to have high numbers of secondary cases (i.e. outbreaks in nurseries and in water secondary/human-contaminated swimming areas). It should be acknowledged, though, that some individuals in outbreaks may be designated as secondary cases because their onset of symptoms is later than that of other cases. This may occur more often when the primary source/mode of transmission is unknown, because cases with primary exposures cannot then be identified. In these circumstances, some individuals who were actually primary cases, who had direct exposure to the (unidentified) source, may simply have developed symptoms at a later date because they had more mature/healthier immune systems, or because they ingested fewer organisms.

However, our study has produced interesting findings, one of the most intriguing being the apparent lack of overall differences between countries in the mean number of confirmed, secondary and primary cases, in median age, or in the rate of secondary cases. The exception was the differences in number of ill cases, and the higher numbers in Japanese outbreaks potentially resulted from broad-based screening of possible contacts. The lack of differences between countries suggests that outbreak size and rate of secondary cases are likely to be determined by epidemiological factors relatively independent of country. This is contrary to what might be expected, given the differences in phage type distributions between contiguous countries[39]. Additionally, research has shown associations between verotoxin profiles and severity of infection[40]. Thus, since verotoxin profiles of phage types tend not to change over time[41], it could be suggested that verotoxin profiles, and thus clinical manifestations of infection could also vary between countries. However, these differences did not manifest in observed differences between countries in our study.

In contrast, our analyses indicate that, unsurprisingly, both mode of secondary transmission and median age are significant determinants in the rate of secondary cases. In particular, outbreaks where the mode of secondary transmission was person to person spread in nurseries had higher numbers of secondary cases and rates of secondary cases. These results corroborate the reported association between outbreaks in nurseries and secondary cases [20], and can be partly explained by the ease of spread between children in nurseries, where close contact of persons with immature immune systems and underdeveloped personal hygiene skills, and a higher likelihood of shedding the bacteria for extended periods of time [42] provide many opportunities for transmission [29]. Children's contact with teachers, parents and siblings then facilitates further transmission, although most cases in nursery outbreaks are the pupils themselves [38,43]. Accompanying higher prevalence rates in young children linked to lower levels of immunity, other studies have shown immunity to pathogenic *E. coli *in populations frequently exposed to farm environments[44,45], with the rate of antibody detection in farm families increasing with age[45]. However, further work, beyond the scope of this paper would be required to fully investigate this impact.

While secondary cases have been noted in primary and secondary school outbreaks included in this study [46,47], secondary transmission in these outbreaks seems limited to family members. This suggests that older pupils may practice hygiene sufficiently [48] to prevent transmission outside close family contact. Also, all the primary/secondary school outbreaks included in this study were thought to be the result of contaminated school food [46,47], while all the nursery outbreaks were suspected to have been triggered by a pupil(s) infected outside the nursery (for example [25,49]).

The interaction between modes of secondary transmission and median age is demonstrated by the fact that, when these two factors are examined together, nursery outbreaks no longer have statistically significantly higher secondary case rates than outbreaks with water as mode of secondary transmission. There was however no change in the relationship between outbreaks where mode of secondary transmission was person to person spread in the home, and outbreaks where mode of secondary transmission was water secondary or person to person spread in a nursery. In addition, lower median age was associated with a higher rate of secondary cases, which corroborates a study where index cases <15 years old were more likely to transmit infection to a household contact, with household contacts aged 1 to 4 years the most likely to become infected[24]. Thus our study appears to confirm, on a wider basis than has been described in other reports or analysed only in outbreaks within single households in a single region/nation[24].

Mode of primary transmission initially appeared to be significant in determining the rate of secondary transmission. However, comparison of analyses including and excluding outbreaks where the primary mode of transmission was unknown revealed that these "source unattributable" outbreaks were responsible for any statistical significance. The higher rate in outbreaks with unknown modes of primary transmission is related to the mode of secondary transmission of these outbreaks, with more than two thirds involving person to person spread in a nursery or recreational water, the categories with the highest rates of secondary cases. When the outbreaks with unknown mode of primary transmission were excluded, outbreaks where water was the mode of primary transmission had higher rates of secondary cases than those spread by food. The occurrence of secondary transmission in more than three quarters of outbreaks, regardless of mode of primary transmission, however, reinforces the need to continue promoting measures to prevent secondary transmission, most importantly hand-washing, as recommended by many other studies. This remains particularly relevant in nurseries, where the drivers of infection are multi-factorial e.g. the immature immunity of the susceptible population, is compounded by their lack of hygienic prevention skills, which is further compounded by the tendency to have substantial numbers of these susceptible individuals in close contact with each other.

## Conclusion

In conclusion, this study provides the first published, international, quantitative description and analysis of secondary cases in *E. coli *O157 outbreaks during the last two decades. Our results indicate that approximately 19% of outbreak cases are secondary, supporting earlier reports that secondary spread within outbreaks is common [20], and suggest that mode of secondary transmission and median age, but not country are important in determining the secondary case characteristics of outbreaks. Outbreaks where mode of secondary transmission was person to person spread in nurseries had higher rates of secondary cases, as did outbreaks where the median age was <6 years. The reasons behind these differences are likely to be multifactorial. Additional research using more detailed, standardised outbreak data, and including other variables such as location and PFGE type would help further elucidate the reasons for some of the differences we have described. This study nonetheless, provides further data to emphasise the importance of simple but effective strategies, such as handwashing, which can reduce the risk of person to person transmission, particularly amongst young children.

## Competing interests

The authors declare that they have no competing interests.

## Authors' contributions

KGS conceived and designed the study, and under the guidance of DJS and RJP carried out the statistical analysis and drafted the manuscript. In addition DJS, RJP and ML extensively modified the manuscript drafts.

## Pre-publication history

The pre-publication history for this paper can be accessed here:

http://www.biomedcentral.com/1471-2334/9/144/prepub

## Supplementary Material

Additional file 1 **Papers and reports referenced in the study**. A listing, by country, of the papers and reports used to obtain information for the 90 outbreaks included in the study. Click here for file
