# Supplementary material for: Primary and secondary cases in Escherichia coli O157 outbreaks: a statistical analysis
Source: BMC Infect Dis. 2009 Aug 28;9:144. doi: 10.1186/1471-2334-9-144 (PMC2741466; doi:10.1186/1471-2334-9-144)
Supplement: Additional file 1 — Papers and reports referenced in the study. A listing, by country, of the papers and reports used to obtain information for the 90 outbreaks included in the study. [file 1471-2334-9-144-S1.doc]

| **Canada** | [1-15] |
| --- | --- |
| **England** | [15-32,32-40] |
| **Finland** | [41] |
| **Ireland** | [42] |
| **Japan** | [43-48] |
| **Scotland** | [49-62] |
| **Sweden** | [63,64] |
| **United States** | [65-95] |
| **Wales** | [95,96] |

Reference List

1. MacDonald DM, Fyfe M, Paccagnella AM, Trinidad A, Louie K, Patrick D: ***Escherichia coli* O157:H7 outbreak linked to salami, British Columbia, Canada, 1999.** *Epidemiology and Infection* 2004, **132:** 283-289.

2. Galanis E, Longmore K, Hasselback P, Swann D, Ellis A, Panaro L: **Investigation of an *E. coli* O157:H7 Outbreak in Brooks, Alberta, June-July 2002: The Role of Occult Cases in the Spread of Infection Within a Daycare Setting.** *Canadian Communicable Disease Report* 2003, **29**.

3. Bruneau A, Rodrigue H, Ismäel J, Dion R, Allard R: **Outbreak of *E. coli* O157 Associated with Bathing at a Public Beach in the Montreal-Centre Region.** *Canadian Communicable Disease Report* 2004, **30**.

4. MacDonald C, Drew J, Carlson R, Dzogan S, Tataryn S, MacDonald AR *et al*.: **Outbreak of *Escherichia coli* O157:H7 leading to the recall of retail ground beef - Winnipeg, Manitba, May 1999.** *Canadian Communicable Disease Report* 2000, **26**.

5. Health Canada. An Outbreak of *Escherichia coli* O157:H7 Infection Associated with Unpasteurized Non-commercial, Custom-pressed Apple Cider - Ontario, 1998. Canadian Communicable Disease Report 25[13]. 1999. 9-10-2004.
Ref Type: Electronic Citation

6. McIntyre L, Fung J, Paccagnella AM, Isaac-Renton J, Rockwell F, Emerson B *et al*.: ***Escherichia coli* O157 Outbreak Associated with the Ingestion of Unpasteurized Goat's Milk in British Columbia, 2001.** *Canadian Communicable Disease Report* 2002, **28**.

7. Sutcliffe P, Picard L, Fortin B, Malaviarachchi D, Hohenadel J, O'Donnell B: ***Escherichia coli* O157:H7 outbreak at a summer hockey camp, Sudbury, 2004.** *Canadian Communicable Disease Report* 2004, **30**.

8. Honish L, Predy G, Hislop N, Chui L, Kowalewska-Grochowska K, Trottier L *et al*.: **An Outbreak of *E. coli* O157:H7 Hemorrhagic Colitis Associated with Unpasteurized Gouda Cheese.** *Canadian Journal of Public Health* 2005, **96:** 182-184.

9. Abbas Z, Balram C, MacDonald BW, Giffin CS, Aramini J, Panaro L: **An Investigation of Two Simultaneous *E. coli* O157:H7 Outbreaks in Health Region 3, New Brunswick, August to September 2003.** *Canadian Communicable Disease Report* 2005, **31:** 229-235.

10. Iebin B, Ison A, Ombos M, Oleszczuk P, Hmed R, Amieson F: **An *E. coli* O157:H7 Outbreak Associated with Consumption of Haggis.**

11. Warshawsky B, Gutmanis I, Henry B, Dow J, Reffle J, Pollett G *et al*.: **Outbreak of *Escherichia coli* O157:H7 related to animal contact at a petting zoo.** *Canadian Journal of Infectious Diseases* 2002, **13:** 175-181.

12. Honish L, Zazulak I, Mahabeer R, Krywiak K, Leyland R, Hislop N *et al*.: **Outbreak of *Escherichia coli* O157:H7 gastroenteritis associated with consumption of beef donairs, Edmonton, Alberta, May-June 2006.** *Canadian Communicable Disease Report* 2007, **33:** 14-20.

13. CDC: **International Notes Outbreak of Hemorrhagic Colitis -- Ottawa, Canada.** *MMWR Weekly* 1983, **32:** 133-134.

14. Lior H: **Hemorrhagic Colitis in a Home for the Aged, Ontario.** *Canada Diseases Weekly Report* 1983, **9:** 29-32.

15. Clark A, Morton S, Wright P, Corkish J, Bolton F, Russell J: **A community outbreak of Vero cytotoxin producing *Escherichia coli* O157 infection linked to a small farm dairy.** *CDR Review* 1997, **7:** R206-R211.

16. Shukla R, Slack R, George A, Cheasty T, Rowe B, Scutter J: ***Escherichia coli* O157 infection associated with a farm visitor centre.** *CDR Review* 1995, **5:** R86-R90.

17. Hildebrand JM, Maguire HC, Holliman RE, Kangesu E: **An outbreak of *Escherichia coli* O157 infection linked to paddling pools.** *CDR Review* 1996, **6:** R33-R36.

18. Goh S, Newman C, Knowles M, Bolton FJ, Hollyoak V, Richards S *et al*.: ***E. coli* O157 phage type 21/28 outbreak in North Cumbria associated with pasteurized milk.** *Epidemiology and Infection* 2002, **129:** 451-457.

19. Allaby MAK, Mayon-White R: ***Escherichia coli* O157: outbreak in a day nursery.** *CDR Review* 1995, **5:** R4-R6.

20. Public Health Laboratory Service: **Outbreak of Vero cytotoxin producing *Escherichia coli* O157 infection in Dorset.** *CDR Weekly* 1998, **8:** 183-186.

21. Public Health Laboratory Service: **Cases of *Escherichia coli* O157 infection associated with unpasteurised cream.** *CDR Weekly* 1998, **8:** 377.

22. Morgan GM, Newman C, Palmer SR, Allen JB, Shepherd W, Rampling A *et al*.: **First recognized community outbreak of haemorrhagic colitis due to verotoxin-producing *Escherichia coli* O157.H7 in the UK.** *Epidemiology and Infection* 1988, **101:** 83-91.

23. Public Health Laboratory Service: **Surveillance of waterborne disease and water quality: July to December 2001.** *CDR Weekly* 2002, **12**.

24. Public Health Laboratory Service: **Two outbreaks of Vero cytotoxin producing *Escherichia coli* O157 infection associated with farms.** *CDR Weekly* 1997, **7:** 263-266.

25. Public Health Laboratory Service: **Outbreak of VTEC O157 infection in East Sussex.** *CDR Weekly* 1999, **9:** 219-222.

26. Public Health Laboratory Service: **Outbreak of VTEC O157 infection at a prison in the Midlands.** *CDR Weekly* 1999, **9:** 281-284.

27. Public Health Laboratory Service: **Outbreaks of VTEC O157 infection linked to comsumption of unpasteurised milk.** *CDR Weekly* 2000, **10:** 203-206.

28. Public Health Laboratory Service: **Two outbreaks of VTEC O157 infection in northern England.** *CDR Weekly* 2000, **10:** 229.

29. Public Health Laboratory Service: **Outbreak of Vero cytotoxin-producing *Escherichia coli* O157 infection in a children's nursery in Suffolk.** *CDR Weekly* 2000, **11**.

30. Crampin M, Willshaw G, Hancock R, Djuretic T, Elstob C, Rouse A *et al*.: **Outbreak of *Escherichia coli* O157 Infection Associated with a Music Festival.** *European Journal of Clinical Microbiology and Infectious Disease* 1999, **18:** 286-288.

31. Gammie AJ, Mortimer PR, Hatch L, Brierley AF, Chada N, Walters JB: **Outbreak of Verocytotoxin-producing *Escherichia coli* O157 associated with cooked ham from a single source.** *PHLS Microbiology Digest* 1996, **13:** 142-145.

32. Health Protection Agency: **Surveillance of waterborne disease outbreaks summary of 2004.** *CDR Weekly* 2006, **16**.

33. Public Health Laboratory Service: **Two contiguous but unconnected outbreaks of Vero cytotoxin-Producing *E. coli* O157 Infection in South East London.** *CDR Weekly* 2006, **15**.

34. Harrison S, Kinra S: **Outbreak of *Escherichia coli* O157 associated with a busy bathing beach.** *Communicable Disease and Public Health* 2004, **7:** 47-50.

35. Health Protection Agency: **VTEC O157 outbreak linked to beach holidays.** *CDR Weekly* 1999, **9:** 327.

36. McDonnell R, Rampling A, Crook S, Cockcroft P, Willshaw G, Cheasty T *et al*.: **An outbreak of Vero cytotoxin producing *Escherichia coli* O157 infection associated with takeaway sandwiches.** *CDR Review* 1997, **7:** R201-R205.

37. Public Health Laboratory Service: **An outbreak of *Escherichia coli* O157 in Southern England.** *CDR Weekly* 1995, **5:** 103.

38. Public Health Laboratory Service: ***Escherichia coli* O157 associated with eating unpasteurised cheese.** *CDR Weekly* 1999, **9:** 113-116.

39. Public Health Laboratory Service: ***Escherichia coli* O157 associated with eating unpasteurised cheese - update.** *CDR Weekly* 1999, **9:** 131-134.

40. Verma A, Bolton FJ, Fiefield D, Lamb P, Woloschin E, Smith N *et al*.: **An Outbreak of *E. coli* O157 associated with a swimming pool: an unusual vehicle of transmission.** *Epidemiology and Infection* 2007.

41. Paunio M, Pebody R, Keskimaki M, Kokki M, Ruutu P, Oinonen S *et al*.: **Swimming-associated outbreak of *Escherichia coli* O157:H7.** *Epidemiology and Infection* 1999, **122:** 1-5.

42. O'Donnell JM, Thornton L, McNamara EB, Prendergast T, Igoe D, Cosgrove C: **Outbreak of Vero cytotoxin-producing *Escherichia coli* O157 in a child day care facility.** *Communicable Disease and Public Health* 2002, **5:** 54-58.

43. Michino H, Araki K, Minami S, Takaya S, Sakai N, Miyazaki M *et al*.: **Massive outbreak of *Escherichia coli* O157 : H7 infection in schoolchildren in Sakai City, Japan, associated with consumption of white radish sprouts.** *American Journal of Epidemiology* 1999, **150:** 787-796.

44. Michino H, Araki K, Minami S, Nakayama T, Ejima Y, Hiroe K *et al*.: **Recent Outbreaks of Infections Caused by *Escherichia coli* O157:H7 in Japan.** In *Escherichia coli O157:H7 and Other Shiga Toxin-Producing E. coli Strains*. Edited by Kaper J, O'Brien A. Washington DC: ASM Press; 1998:73-82.

45. Sugiyama A, Iwade Y, Akachi S, Nakano Y, Matsuno Y, Yano T *et al*.: **An Outbreak of Shigatoxin-Producing *Escherichia coli* O157:H7 in a Nursery School in Mie Prefecture.** *Japanese Journal of Infectious Diseases* 2005, **58:** 398-400.

46. Maruzumi M, Morita M, Matsuoka Y, Uekawa A, Nakamura T, Fuji K: **Mass Food Poisoning by Beef Offal Contaminated by *Escherichia coli* O157.** *Japanese Journal of Infectious Diseases* 2005, **58:** 397.

47. Terajima J, Izumiya H, Iyoda S, Tamura K, Watanabe H: **Detection of a multi-prefectural *E. coli* O157:H7 outbreak caused by contaminated Ikura-Sushi ingestion.** *Japanese Journal of Infectious Diseases* 1999, **52:** 52-53.

48. Yamamoto J, Ishikawa A, Miyamoto M, Nomura T, Uchimura M, Koiwai K: **Outbreak of enterohemorrhagic *Escherichia coli* O157 mass infection caused by 'whole roasted cow'.** *Japanese Journal of Infectious Diseases* 2001, **54:** 88-89.

49. Cowden JM, Ahmed S, Donaghy M, Riley A: **Epidemiological investigation of the Central Scotland outbreak of *Escherichia coli* O157 infection, November to December 1996.** *Epidemiology and Infection* 2001, **126**.

50. Licence K, Oates KR, Synge BA, Reid TMS: **An outbreak of *E. coli* O157 infection with evidence of spread from animals to man through contamination of a private water supply.** *Epidemiology and Infection* 2001, **126:** 135-138.

51. Coia JE, Davis B, Reilly W: ***E. coli* O157 infection in Scotland, 1994.** *SCIEH Weekly Report* 1996, **30:** 29-30.

52. Howie H, Mukerjee A, Cowden JM, Leith J, Reid TMS: **Investigation of an outbreak of *Escherichia coli* O157 infection caused by environmental exposure at a scout camp.** *Epidemiology and Infection* 2003, **131:** 1063-1069.

53. Marsh J, MacLeod AF, Hanson M, Emmanuel FXS, Frost J, Thomas A: **A restaurant-associated outbreak of *E. coli* O157 infection.** *Public Health* 1992, **14:** 78-83.

54. Brewster DH, Brown MI, Robertson D, Houghton GL, Bimson J, Sharp JCM: **An outbreak of *Escherichia coli* O157 associated with a children's paddling pool.** *Epidemiology and Infection* 1994, **112:** 441-447.

55. Kohli HS, Chaudhuri AKR, Todd WTA, Mitchell AAB, Liddell KG: **A Severe Outbreak of *Escherichia coli* O157 in 2 Psychogeriatric Wards.** *Journal of Public Health Medicine* 1994, **16:** 11-15.

56. SCIEH: **Outbreak of *Escherichia coli* O157 Infection in Greater Glasgow.** *SCIEH Weekly Report* 1998, **35:** 191.

57. SCIEH: ***E. coli* outbreak in Grampian.** *SCIEH Weekly Report* 1999, **33:** 156.

58. Jones IG, Roworth M: **An outbreak of *Escherichia coli* 0157 and campylobacteriosisassociated with contamination of a drinking water supply.** *Public Health* 1996, **110:** 277-282.

59. SCIEH: ***E. coli* O157 outbreak associated with a garden centre.** *SCIEH Weekly Report* 2001, **35:** 245.

60. O'Brien S, Murdoch PS, Riley A, King I, Barr M, Murdoch S *et al*.: **A foodborne outbreak of Vero cytotoxin-producing *Escherichia coli* O157:H-phage type 8 in hospital.** *Journal of Hospital Infection* 2001, **49:** 167-172.

61. Upton PA, Coia JE: **Outbreak of *Escherichia coli* O157 infection associated with pasteurised milk supply.** *The Lancet* 1994, **344:** 1015.

62. Davis B, Brogan R: **A widespread community outbreak of *E. coli* O157 infection in Scotland.** *Public Health* 1995, **109:** 381-388.

63. Wahl M, Andersson Y: **EHEC cases from an international football tournament (Gothia Cup) in Sweden, July 2004.** *Eurosurveillance Weekly* 2004, **8**.

64. Welinder-Olsson C, Stenqvist K, Badenfors M, Brandberg A, Floren K, Holm M *et al*.: **EHEC outbreak among staff at a children's hospital - use of PCR for verocytotoxin detection and PFGE for epidemiological investigation.** *Epidemiology and Infection* 2004, **132:** 43-49.

65. Breuer T, Benkel D, Shapiro R, Hall W, Winnett M, Linn M *et al*.: **A Multistate Outbreak of *Escherichia coli* O157:H7 Infections Linked to Alfalfa Sprouts Grown from Contaminated Seeds.** *Emerging Infectious Diseases* 2001, **7:** 977-982.

66. Gage R, Crielly A, Baysinger M, Chernak E: **Outbreaks of *Escherichia Coli* O157:H7 Infections Among Children Associated with Farm Visits - Pernnsylvania and Washington, 2000.** *MMWR Weekly* 2001, **50:** 293-97.

67. Crump J, Braden C, Dey M, Hoekstra R, Rickelman-Apisa J, Baldwin D *et al*.: **Outbreaks of *Escherichia coli O157* infections at multiple county agricultural fairs: a hazard of mixing cattle, concession stands and children.** *Epidemiology and Infection* 2003, **131:** 1055-1062.

68. Keene WE, Hedberg K, Herriott DE, Hancock DD, McKay RW, Barrett T *et al*.: **A Prolonged Outbreak of *Escherichia coli* O157:H7 Infections Caused by Commercially Distributed Raw Milk.** *The Journal of Infectious Diseases* 1997, **176:** 815-818.

69. Hilborn ED, Mermin JH, Mshar P, Hadler J, Voetsch AC, Wojtkunski C *et al*.: **A Multistate Outbreak of *Escherichia coli* O157:H7 Infections Associated With Consumption of Mesclun Lettuce.** *Archives of Internal Medicine* 1999, **159:** 1758-1764.

70. Belongia EA, Osterholm MT, Soler JT, Ammend DA, Braun JE, MacDonald KL: **Transmission of *Escherichia coli* O157:H7 Infection in Minnesota Child Day-care Facilities.** *Journal of the American Medical Association* 1993, **269:** 883-888.

71. Keene WE, Mcanulty JM, Hoesly FC, Williams LP, Hedberg K, Oxman GL *et al*.: **A Swimming-Associated Outbreak of Hemorrhagic Colitis Caused by *Escherichia coli* O157:H7 and Shigella-Sonnei.** *New England Journal of Medicine* 1994, **331:** 579-584.

72. Friedman MS, Roels T, Koehler JE, Feldman L, Bibb WF, Blake P: ***Escherichia coli* O157:H7 outbreak associated with an improperly chlorinated swimming pool.** *Clinical Infectious Diseases* 1999, **29:** 298-303.

73. Cody SH, Glynn MK, Farrar JA, Cairns KL, Griffin PM, Kobayashi J *et al*.: **An Outbreak of *Escherichia coli* O157:H7 Infection from Unpasteurized Commercial Apple Juice.** *Ann Intern Med* 1999, **130:** 202-209.

74. Belongia EA, Macdonald KL, Parham GL, White KE, Korlath JA, Lobato MN *et al*.: **An Outbreak of *Escherichia coli* O157:H7 Colitis Associated with Consumption of Precooked Meat Patties.** *Journal of Infectious Diseases* 1991, **164:** 338-343.

75. Spika JS, Parsons JE, Nordenberg D, Wells JG, Gunn RA, Blake PA: **Hemolytic Uremic Syndrome and Diarrhea Associated with *Escherichia coli* O157:H7 in A Day-Care-Center.** *Journal of Pediatrics* 1986, **109:** 287-291.

76. Ostroff SM, Griffin PM, Tauxe RV, Shipman LD, Greene KD, Wells JG *et al*.: **A Statewide Outbreak of *Escherichia coli* O157:H7 Infections in Washington-State.** *American Journal of Epidemiology* 1990, **132:** 239-247.

77. Banatvala N, Magnano AR, Cartter ML, Barrett TJ, Bibb WF, Vasile LL *et al*.: **Meat Grinders and Molecular Epidemiology: Two Supermarket Outbreaks of *Escherichia coli* O157:H7 Infection.** *Journal of Infectious Diseases* 1996, **173:** 480-483.

78. Centers for Disease Control and Prevention: ***Escherichia coli* O157:H7 Outbreak Linked to Home-Cooked Hamburger - - California, July 1993.** *MMWR Weekly* 1994, **43:** 213-216.

79. Centers for Disease Control and Prevention: **Outbreaks of *Escherichia coli* O157:H7 Associated with Petting Zoos --- North Carolina, Florida, and Arizona, 2004 and 2005.** *MMWR Weekly* 2005, **54:** 1277-1280.

80. Ackman D, Marks S, Mack P, Caldwell M, Root T, Birkhead G: **Swimming-associated haemorrhagic colitis due to *Escherichia coli* O157:H7 infection: evidence of prolonged contamination of a fresh water lake.** *Epidemiology and Infection* 1997, **119:** 1-8.

81. Feldman KA, Mohle-Boetani JC, Ward J, Furst K, Abbott SL, Ferrero DV *et al*.: **A cluster of *Escherichia coli* O157: nonmotile infections associated with recreational exposure to lake water.** *Public Health Reports* 2002, **117:** 380-385.

82. Centers for Disease Control and Prevention: ***Escherichia coli* O157:H7 Outbreak Linked to Commercially Distributed Dry-Cured Salami -- Washington and California, 1994.** *MMWR Weekly* 1995, **44:** 157-160.

83. Gouveia S, Proctor M, Lee M-S, Luchansky JB, Kaspar CW: **Genomic Comparisons and Shiga Toxin Production among *Escherichia coli* O157:H7 Isolates from a Day Care Center Outbreak and Sporadic Cases in Southeastern Wisconsin.** *Journal of Clinical Microbiology* 1998, **36:** 727-733.

84. Ferguson DD, Scheftel J, Cronquist A, Smith K, Woo-Ming A, Anderson E *et al*.: **Temporally distinct *Escherichia coli* O157 outbreaks associated with alfalfa sprouts linked to a common seed source - Colorado and Minnesota, 2003.** *Epidemiology and Infection* 2005, **133:** 439-447.

85. Proctor ME. Investigation of an Outbreak of *E. coli* O157:H7 Infection at the Layton Avenue Sizzler Restaurant Associated with Cross-Contamination of Watermelon with Raw Meat, Milwaukee, WI; July - August, 2000. 29-9-2000. Communicable Disease Epidemiology Section, Bureau of Communicable Diseases, Wisconsin Division of Public Health, Department of Health and Family Services (Wisconsin).
Ref Type: Report

86. Bhat M, Denny J, MacDonald K, Hofmann J, Jain S, Lynch M: ***Escherichia coli* O157:H7 Infection Associated with Drinking Raw Milk - Washington and Oregon, November-December 2005.** *MMWR Weekly* 2007, **56:** 165-167.

87. Proctor ME. Investigation of an Outbreak of Gastrointestinal Illness at the Mayfair Road Sizzler Restaurant, Wauwatosa, WI: July - August 2000. 9-10-2000. Department of Health and Family Servicesm, Wisconsin Division of Public Health.
Ref Type: Report

88. Bell B, Goldoft M, Griffin PM, Davis M, Gordon D, Tarr P *et al*.: **A Multistate Outbreak of *Escherichia coli* O157:H7 - Associated Bloody Diarrhea and Hemolytic Uremic Syndrome From Hamburgers.** *Journal of the American Medical Association* 1994, **272:** 1349-1353.

89. Tuttle J, Gomez T, Doyle MP, Wells JG, Zhao T, Tauxe RV *et al*.: **Lessons from a large outbreak of *Escherichia coli* O157:H7 infections: insights into the infectious dose and method of widespread contamination of hamburger patties.** *Epidemiology and Infection* 1999, **122:** 185-192.

90. Bruce MG, Curtis MB, Payne MM, Gautom RK, Thompson EC, Bennett AL *et al*.: **Lake-associated outbreak of *Escherichia coli* O157:H7 in Clark County, Washington, August 1999.** *Archives of Pediatrics & Adolescent Medicine* 2003, **157:** 1016-1021.

91. Samadpour M, Stewart J, Steingart K, Addy C, Louderback J, McGinn M *et al*.: **Laboratory Investigation of an *E. coli* O157:H7 OUtbreak Associated with Swimming in Battle Ground Lake, Vancouver, Washington.** *Journal of Environmental Health* 2002, **64:** 16-20.

92. Reiss G, Kunz P, Koin D, Keeffe EB: ***Escherichia coli* O157 : H7 infection in nursing homes: Review of literature and report of recent outbreak.** *Journal of the American Geriatrics Society* 2006, **54:** 680-684.

93. San Mateo County Health Services Agency: **Outbreak Investigation of *Escherichia coli* O157:H7.** *Epidemiological Bulletin - San Mateo County Health Services Agency* 2004, **3:** 6-7.

94. Palumbo MS, Sigl J, Farrar JA, Waddell JM. Investigation of *E. coli* O157:H7 Outbreak at San Mateo County Retirement Facility. California Department of Health Services Website . 3-5-2004. State of California - Health and Human Services Agency, Department of Health Services, Food and Drug Branch.
Ref Type: Electronic Citation

95. Payne CJI, Petrovic M, Roberts RJ, Paul A, Linnane E, Walker M *et al*.: **Vero cytotoxin-producing *Escherichia coli* O157 gastroenteritis in farm visitors, North Wales.** *Emerging Infectious Diseases* 2003, **9:** 526-530.

96. Public Health Laboratory Service: **VTEC O157 outbreak associated with a farm visitor centre in North Wales.** *CDR Weekly* 1999, **9:** 227-230.
